# Development and psychometric evaluation of a new patient -reported outcome measure for psoriasis self-management efficacy: the self-management efficacy questionnaire among patients with psoriasis (SMEQ-PSO)

**DOI:** 10.1186/s12955-023-02134-w

**Published:** 2023-06-06

**Authors:** Yuanhui Sun, Xiujie Zhang, Zhen Yang, Aiping Wang

**Affiliations:** 1 Department of Public Service, The First Affiliated Hospital of China Medical University, Shenyang, Liaoning Province China; 2 Department of Cardiology, The First Affiliated Hospital of Dalian Medical University, Dalian, Liaoning Province China; 3 The First Affiliated Hospital of China Medical University, No.155, Nanjing North Street, Heping District, Shenyang, Liaoning Province China

**Keywords:** Psoriasis, Questionnaire, Self-management, Self- efficacy, Psychometric validation

## Abstract

**Background:**

It is significant for the healthy outcome of patients with psoriasis (PSO) to improve their self-management efficacy. A standardized assessment tool, however, was lacking. Therefore, we aimed to develop a self-management efficacy questionnaire for patients with PSO (SMEQ-PSO) and evaluate its psychometric properties.

**Methods:**

A cross-sectional study developing clinical evaluation tool was conducted from October 2021 to August 2022. In the process of developing SMEQ-PSO, three phases were involved: item generation, item evaluation, and psychometric evaluation.

**Results:**

The SMEQ-PSO with five dimensions and 28 items was developed. The questionnaire’s content validity index was 0.976. Exploratory factor analysis indicated a five-factor structure (self-efficacy of psychosocial adaptation, self-efficacy of daily life management, self-efficacy of skin management, self-efficacy of disease knowledge management and self-efficacy of disease treatment management) that explained 62.039% of the total variance. Confirmatory factor analysis indicated appropriate fit of the five-factor model. The overall Cronbach’α coefficient was 0.930, the test-retest reliability was 0.768 and the split half reliability coefficients was 0.952.

**Conclusions:**

The 28-item SMEQ-PSO is a reliable and valid tool that can be used to assess the self-management efficacy among patients with PSO and provide personalized interventions based on their individual circumstances to improve their health outcomes.

## Background

Psoriasis (PSO) is an immune-mediated chronic, recurrent, inflammatory, systemic disease induced by the combination of heredity and environment, commonly characterized by development of erythematous, indurated, scaly, pruritic and commonly painful skin plaques [1]. The global prevalence of PSO is currently 0.09%-11.43%, with approximately 125 million people affected, making it a global public health problem [2]. Based on the Global Burden of Disease (GBD) study, the incidence of PSO showed an increasing trend, and it has attracted more attention [3]. Patients with PSO usually suffer from symptoms related to the skin, but mounting epidemiological and basic scientific studies have found that PSO is not only limited to the skin, but can also be complicated by a variety of systemic and metabolic diseases [4]. 73% of patients with PSO have at least one comorbidity with PSO, such as joint damage, cardiovascular disease, mental-emotional disorders, metabolic syndrome, and tumors, which can significantly reduce a patient’s life expectancy and negatively impact their prognosis [5]. In addition, due to protracted course of disease, repeated outbreak and disfiguring, PSO often causes multiple burdens such as physical, psychological, economic and social burdens, which can have a significant influence on a patient’ s health-related quality of life [6]. Numerous studies have shown that PSO has similar impacts on patients’ quality of life to other chronic diseases, even though its physiological effects are not as severe as those of chronic diseases as cancer, diabetes, or cardiovascular disease [7].

PSO is highly susceptible to intrinsic and extrinsic risk factors including mental stress, drugs, infections, unhealthy lifestyle (such as obesity, smoking and alcohol) which means that patients necessitates long-term coexistence with the disease and the practice of scientific and effective self-management [8]. Furthermore, global psoriasis treatment guidelines emphasize the importance of disease self-management [9–12]. As a major component of PSO care, self-management is critical, and the implementation of systematic and comprehensive self-management programs will result in better disease control and a higher quality of life for patients with PSO [13, 14]. However, the current state of disease self-management in patients with PSO is unsatisfactory, primarily owing to poor medication adherence and inadequate management of non-drug factors [15]. Previous studies have focused on medication adherence, but it has been overlooked that non-drug factors can also lead to disease recurrence, exacerbation, and an increased risk of hospitalization [16, 17]. As a result, patients with PSO should take the initiative to strengthen long-term comprehensive management including diet, exercise, lifestyle, skin care, and emotion, in addition to actively cooperating with disease-related treatment [18].

As a key determinant of effective chronic disease management, self-management efficacy (SME) is based on Bandura’s self-efficacy theory, evolved from self-efficacy in the context of self-management [19], and expressed as patients’ beliefs or confidence in their ability to successfully deal with and manage their own illness, and can directly or indirectly contribute to the implementation of health behaviors through behavioral management and emotional management [20, 21]. This study defined self-management efficacy among patients with PSO as patients’ confidence or belief in their capacity to successfully control disease progression, relieve clinical symptoms, prevent or reduce disease recurrence, control the comorbidities associated with PSO and enhance quality of life while undergoing disease therapy and management [19, 22]. Patients with a higher level of self-management efficacy are able to effectively manage the disease in a positive manner. Nevertheless, in patients with PSO, self-management efficacy is generally low [23], and there is a close relationship between self-management efficacy and quality of life [24]. Thus, in order to better understand the current state of self-management among patients with PSO and direct health professionals in further providing personalized interventions to improve health outcomes, it is vital to evaluate the level of self-management efficacy. As of yet, no appropriate evaluation instruments have been developed to measure self-management efficacy among patients with PSO. Hence, the purpose of our study was to develop a self-report questionnaire to assess the level of self-management efficacy among patients with PSO and evaluate its psychometric properties.

## Methods

### Participants

Eligible participants were recruited by convenience sampling in two tertiary hospitals in Shenyang, China from October 2021 to August 2022. Inclusion criteria were: (a) age of 18 years or above, (b) diagnosed with psoriasis, (c) fluent in Chinese both orally and in writing, and (d) voluntary to participate in the study and provided the informed consent. Participants suffering from severe cognitive disorders, cardiovascular diseases or other diseases which of serious effects on their quality of life, and consolidated with other skin diseases were excluded. The general guideline of factor analysis determined that the sample size should be five to ten participants for each item with at least 200 cases [25]. In this study, the initial questionnaire consisted of 43 items, and a total of 440 patients with psoriasis were finally recruited.

### Study design

**Fig. 1:**
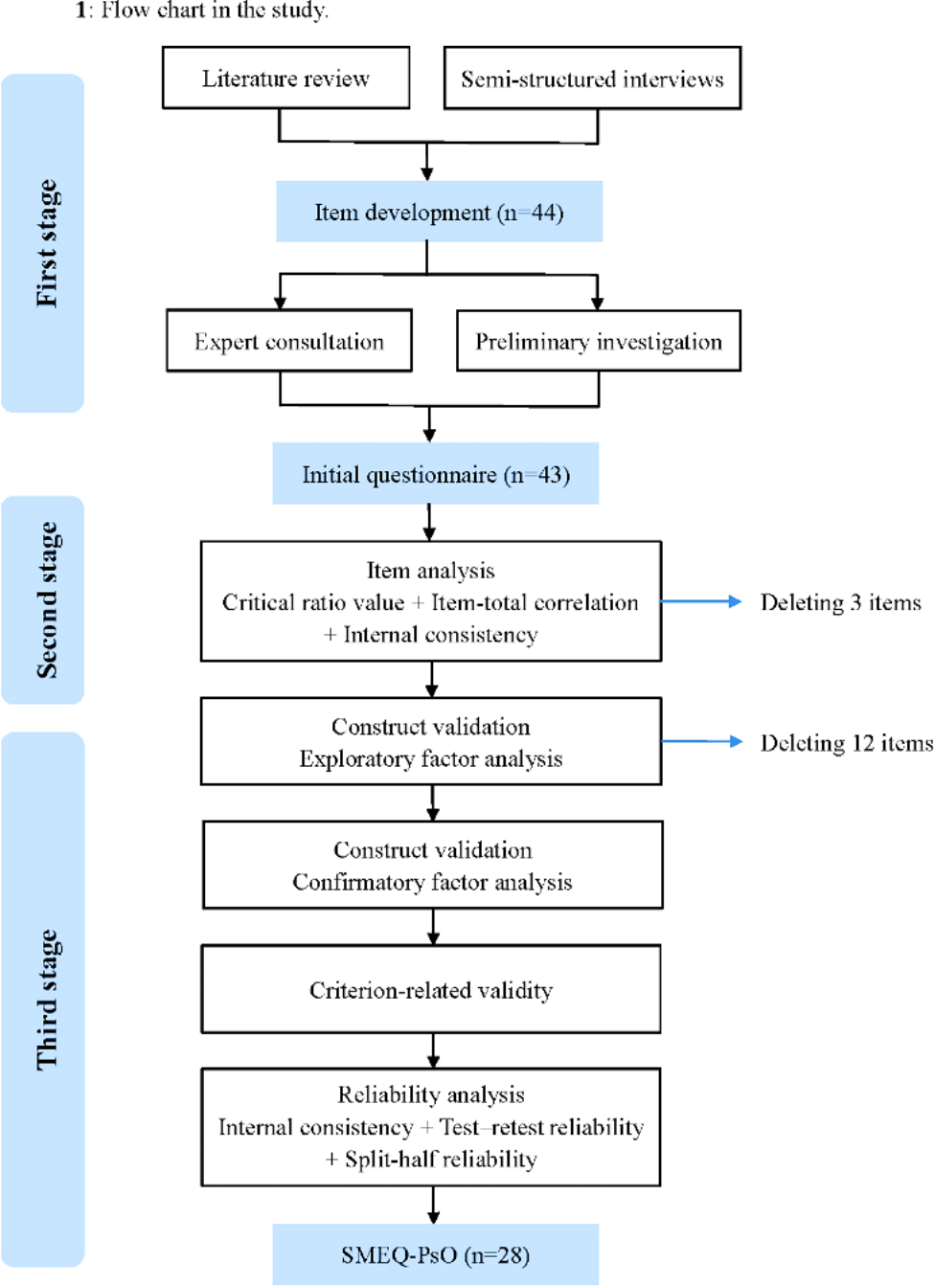
Flow chart in the study

A cross-sectional study developing clinical evaluation tool was conducted from October 2021 to August 2022. The development of this questionnaire followed three phases: (a) item generation, (b) item evaluation, and (c) psychometric evaluation of questionnaire (Fig. 1) [26].

### Item generation

According to self-efficacy theory, the conceptual for the self-management efficacy evolved from self-efficacy in the context of self-management [19]. To develop the items for the SMEQ-PSO, we conducted a comprehensive literature review related to the self-management efficacy in patients with PSO [27]. The initial draft of the SMEQ-PSO was based on the following: guidelines of the Chinese Society of Dermatology [22], Joint AAD-NPF guidelines of care for the management and treatment of psoriasis with awareness and attention to comorbidities [9], Consensus guidelines for the management of plaque psoriasis [28], guidelines of Psoriasis: assessment and management [12], Asian consensus on assessment and management of mild to moderate plaque psoriasis with topical therapy [10]. Furthermore, based on the findings from the review, drawing on the item pool of other chronic disease self-management scales to supplement with items, and semi-structured interviews were conducted with patients with PSO to comprehensively understand their self-management status and related needs. A total of 7 experts were invited to revise the initial draft of the SMEQ-PSO. Inclusion criteria of experts were: (a) at least 10 years in the field of expertise, (b) at least intermediate title, (c) at least master degree, and (d) voluntary participants in the study. They were asked to consider whether the questionnaire covered all aspects of self-management in patients with PSO, and provide feedback and suggestions for improving it. To test the accuracy of language and readability of the SMEQ-PSO, ten PSO patients who met the inclusion and exclusion criteria completed the questionnaire in a pre-study and were asked to comment on any unclear items. The item pool was formed with 44 items. The questionnaire had a five-point Likert-type format with responses as “completely unconfident” (1), “less confident” (2), “confident” (3), “quite Confident” (4), and “absolutely completely confident” (5). Higher scores indicate better self-management efficacy.

### Item evaluation

The quality of the item was estimated by the item analysis with the critical ratio (CR) value, item-total correlation (ITC) and internal consistency. The CR was arranging the total score of the questionnaire from low to high, according to the critical score of 27%, the subjects were divided into a low group and a high group. After t-test for two independent samples, the items whose CR value<3 were deleted [29]. The ITC analysis was used to remove items with results<0.4 [29]. In addition, the Cronbach’s α coefficient after deleting each item was calculated to determine the homogeneity of the item. If the Cronbach’s coefficient of the entire questionnaire was significantly higher after deleting an item, it was considered for deletion [29].

### Psychometric evaluation

#### Content validity

A total of seven experts were invited to evaluate the content validity of the questionnaire supported by the content validity index of the item (I-CVI) and the content validity index of the scale (S-CVI). I-CVI is the ratio of the number of experts who ranked each item by three point or four point to the total number of experts. S-CVI is the mean of I-CVI for all items. The requirements were as follows: (a) I-CVI was 0.78 and above and (b) S-CVI was 0.90 and above [30].

### Construct validity

Construct validity was evaluated by factor analysis, including exploratory factor analysis (EFA) and confirmatory factor analysis (CFA). According to the factor loading value and item content in the EFA, the underlying factor structure of the questionnaire were determined. The CFA was completed by structural equation modeling in this study on the basis of EFA to verify the stability of the structure of the SMEQ-PSO. Additionally, convergent validity and discriminant validity were performed to evaluate the questionnaire’s construct validity. For convergent validity, the average variance extracted (AVE) value and the composite reliability (CR) value were used. The AVE value is greater than 0.50 and the CR value is greater than 0.80, indicating that the questionnaire has adequate convergent validity [31]. For discriminant validity, the square root of the AVE value and the factor correlation coefficient were calculated. We require that the square root of the AVE value exceed the correlation coefficient between the corresponding factors, and that the correlations be significant (*P*<0.001) among the factors [31].

### Criterion validity

The General Self-Efficacy Scale was adopted as a criterion instrument to appraise the criterion validity of the SMEQ-PSO, as there are no other self-management tools for patients with PSO. The General Self-Efficacy Scale (GSES) was developed by Schwarzer et al. [32] in Germany. This scale contains one dimension with 10 items, answered on a 4-point scale. The higher the score, the higher the sense of self-efficacy(Cronbach’s α = 0.87).

### Reliability analysis

The reliability of the SMEQ-PSO was assessed with internal consistency reliability, test-retest reliability, and split-half reliability. Cronbach’s α is frequently used to evaluate a questionnaire’s internal consistency. The Cronbach’s α coefficient and split-half reliability coefficient were calculated to evaluate item’s homogeneity. Test-retest reliability reflects the stability of the questionnaire. After 2 weeks, 25 patients completed the questionnaire again, and the correlation coefficient between the two measurements was calculated to evaluate test-retest reliability. We demand that the Cronbach’α coefficient, the split-half reliability coefficient and test–retest reliability coefficient should all be at least 0.7 [33, 34].

### Data analysis

SPSS 26.0 and AMOS 24.0 were used for data analysis. Frequency and percentage were adopted to describe the sociodemographic and clinical characteristics of the study population. Item analysis was completed to evaluate and screen the items, and the expert consultation was adopted to assess the content validity of the questionnaire. Construct validity was assessed by factor analysis, including EFA and CFA. The General Self-Efficacy Scale (GSES)was used as the correlation validity of the calibration test questionnaire. The internal consistency analysis, test–retest reliability analysis and split-half reliability analysis were employed to assess the homogeneity and stability of the questionnaire.

## Results

### Participant characteristics

A total of 452 participants were recruited, and 440 questionnaires were finally valid (effective response rate = 97.35%). Among them, 62.3% were male, 66.8% were married, and 63.9% were employed. The age of the participants ranged from 18 to 83 years, and the mean age was 42.78 ± 14.45 years; the mean course of disease was 15.68 ± 11.49 years. Participants’ sociodemographic and clinical characteristics are shown in Table 1.

**Table 1:** Sociodemographic and clinical characteristics of the participants(n = 440)

Characteristics	N (%)
Gender	
Male	274(62.3%)
Female	166(37.7%)
Age(years)	
≤ 60	384 (87.3%)
>60	56 (12.7%)
Education level	
Secondary school or below	115 (26.1%)
Vocational/ High school	86 (19.5%)
Undergraduate college or above	239 (54.3%)
Employment situation	
Working	281 (63.9%)
Unemployed	79 (17.9%)
Retired	69 (15.7%)
Others	11 (2.5%)
Marital status	
Married	294 (66.8%)
Unmarried	114 (25.9%)
Divorced	24 (5.5%)
Widowed	8 (1.8%)
Smoking	
Yes	132 (30.0%)
No	308 (70.0%)
Drinking	
Yes	106 (24.1%)
No	334 (75.9%)
Course of disease(years)	
<1	36 (8.2%)
1 ~ 10	148 (33.6%)
>10	256(58.2%)
Itching	
Yes	357(81.1%)
No	83 (18.9%)
Pain	
Yes	211(48.0%)
No	229(52.0%)
Body surface area (BSA)	
<3%	100(22.7%)
3%~<10%	183(41.6%)
≥ 10%	157(35.7%)
Comorbidity	
Yes	121(27.5%)
No	319(72.5%)

### Item generation

After the literature review and semi-structured interviews, the initial draft of the SMEQ-PSO contained 44 items, which were developed from the literature (31 items) and qualitative research (13 items). Seven experts (3 dermatologists and 4 nursing specialists) assessed the content validity of the questionnaire and revised the draft of SMEQ-PSO, and the expert response rate was 100%. According to expert evaluation, except for items 10, 29 and 31, whose I-CVI were 0.71, the I-CVI of other items were above 0.80, and S-CVI of the questionnaire was 0.95. Based on item deletion criteria and experts’ comments, following group discussion, 3 items were deleted, 7 items were modified, and 2 items were added. After a preliminary survey using the revised draft of the SMEQ-PSO, all 10 patients with psoriasis reported that each item of the questionnaire was easy to understand, and they could answer based on their actual situation. The initial questionnaire with 43 items was finally developed.

### Item evaluation

Item analysis was performed using CR, ITC and Cronbach’s α coefficient. In this study, CR value of the items ranged from 2.537 to 15.593. Item 5 did not meet the criteria, and was deleted. ITC coefficient ranged from 0.205 to 0.701. Items with ITC<0.4 including items 3, 5, and 19 were also deleted. The Cronbach’s α coefficient increased after item 5 was deleted. After the item evaluation, the SMEQ-PSO contained 40 items. The detailed information is shown in Table 2.

**Table 2:** Item analysis

Item	CR	Corrected Item-total correlation coefficients	Cronbach’s α coefficient if item deleted
1	7.147	0.494	0.947
2	7.647	0.502	0.947
3	4.705	0.310^*^	0.948
4	7.335	0.542	0.946
5	2.537^*^	0.205^*^	0.949^*^
6	7.703	0.551	0.946
7	9.673	0.589	0.946
8	11.228	0.599	0.946
9	7.293	0.413	0.947
10	12.94	0.591	0.946
11	9.101	0.414	0.947
12	13.537	0.506	0.947
13	11.098	0.57	0.946
14	11.121	0.506	0.947
15	9.512	0.620	0.946
16	9.178	0.579	0.946
17	7.664	0.484	0.947
18	12.850	0.586	0.946
19	5.162	0.265^*^	0.948
20	15.593	0.549	0.946
21	12.492	0.610	0.946
22	14.259	0.629	0.946
23	9.622	0.477	0.947
24	8.553	0.491	0.947
25	12.229	0.570	0.946
26	8.500	0.427	0.947
27	13.264	0.578	0.946
28	12.869	0.670	0.946
29	14.714	0.701	0.945
30	12.099	0.645	0.946
31	9.339	0.558	0.946
32	11.685	0.583	0.946
33	11.757	0.536	0.946
34	13.547	0.642	0.946
35	12.974	0.563	0.946
36	14.011	0.594	0.946
37	10.355	0.629	0.946
38	11.819	0.597	0.946
39	10.325	0.492	0.947
40	9.046	0.526	0.946
41	10.074	0.671	0.946
42	10.056	0.671	0.946
43	9.397	0.548	0.946
Criteria	≥ 3	≥ 0.4	≤ 0.948

Note: ^*^ indicates that the item was excluded by the corresponding method

### Psychometric evaluation

#### Content validity

As a result of expert consultation, the content validity index of the questionnaire was 0.976, and the content validity index of all items ranged from 0.80 to 1.00.

### Construct validity

The value of Kaiser-Meyer-Olkin (KMO) was 0.905 and the result of Bartlett’s sphericity test was 4151.542 (*P*<0.001), indicating that the items of the questionnaire were suitable for factor analysis. The cumulative explained variance rate of the five-factor model was 62.039% with eigenvalues > 1, and the item factor loadings was from 0.453 to 0.831(Table 3), which meet requirements as follows:(1) the factor loading of the item ≥ 0.4, with no cross loading, and (2) each extracted common factor contains at least three items. Through five rounds of EFA, a total of 12 items were deleted from the questionnaire, which contained 28 items. Based on the content expressed by the item, five factors were created and labelled as: self-efficacy of psychosocial adaptation (8 items), self-efficacy of daily life management (8 items), self-efficacy of skin management (5 items), self-efficacy of disease knowledge management (4 items) and self-efficacy of disease treatment management (3 items).

**Fig. 2:**
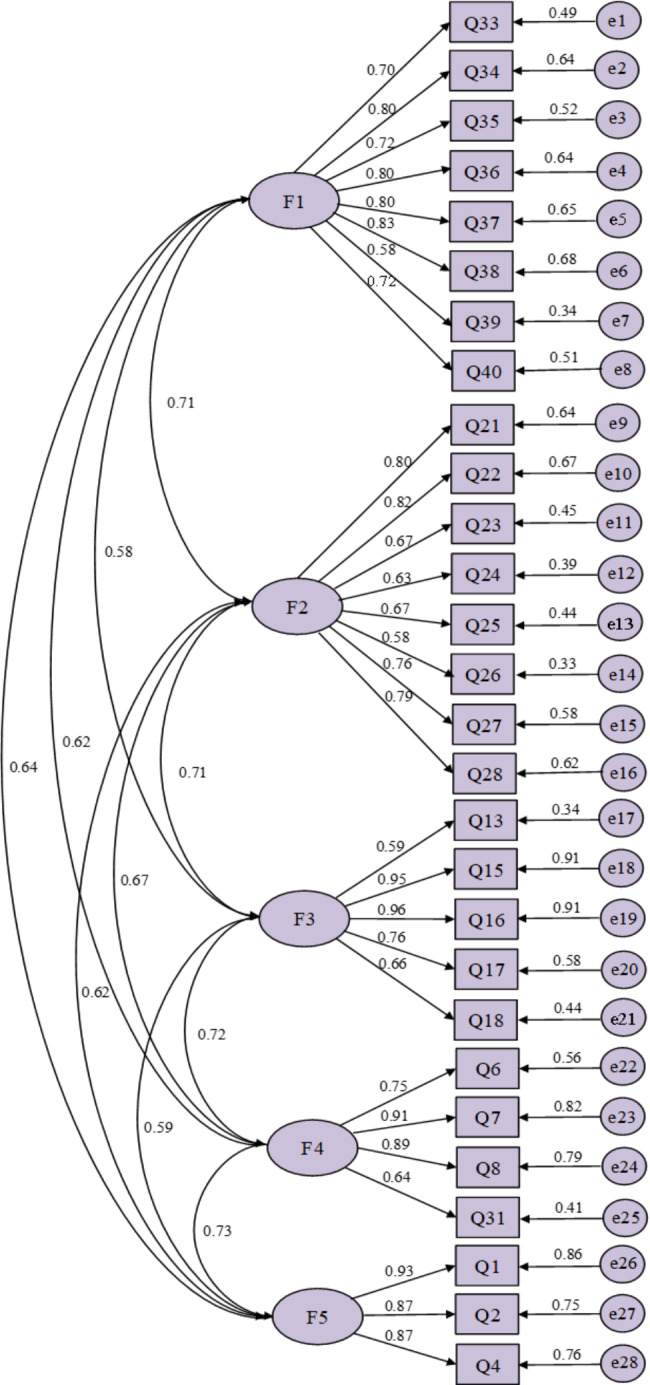
The standardized estimate of each coefficient in the confirmatory factor analysis model

To test the explored five-factor model, the 28 items were subjected to the CFA. As the results of CFA, the chi-square degree of freedom( χ2/df ) = 2.418, the root mean square error of approximation (RMSEA) = 0.074, the goodness-of-fit index (GFI) = 0.817, the comparative fit index (CFI) = 0.908, the incremental fit index (IFI) = 0.909, the parsimonious normed-of-fit index (PNFI) = 0.768, and the parsimonious goodness-of-fit index (PGFI) = 0.684, all of which fell in the acceptable range. The selected fitting indexes indicated that the five-factor model obtains a good degree of fitness. Thus, no items were removed from the CFA. In addition, in convergent validity analysis, the AVE values ranged from 0.515 to 0.789, all greater than 0.5, and the CR values ranged from 0.858 to 0.925, all greater than 0.8. In discriminant validity analysis, the square root values of AVE ranged from 0.718 to 0.888, all of which were greater than the correlation coefficient between the respective factors. The results are shown in Tables 4 and 5; Fig. 2.

**Table 3:** Item Factor Loadings

		Item	Factor 1		Factor 2		Factor 3	Factor 4	Factor 5
35		When I am unable to release negative emotions on my own, I can talk to others.	0.792						
36		I can actively express my thoughts and feelings with my family during treatment.	0.788						
38		When I am in distress, I can seek consolation and assistance from my family or friends.	0.771						
40		I can actively participate in daily social and collective activities	0.758						
34		When I’m feeling down, I can brighten myself up.	0.699						
33		I can keep a positive and optimistic attitude during my illness.	0.686						
37		I can maintain family functions such as taking care of myself and my family, participating in family activities, and so on.	0.665						
39		I can take the initiative to meet ward mates and establish good communication and exchange with them.	0.597						
27		I can try to strike a balance between work and rest in daily life.			0.738				
23		I can keep my weight under reasonable control.			0.688				
25		I can carry out suitable physical exercise. (Focus on take aerobic exercise, such as walking/jogging/taijiquan?)			0.682		-		
22		I can maintain a regular diet and avoid overeating or excessive hunger.			0.645				
26		I can keep regular hours and get plenty of sleep.			0.630				
21		I can keep a reasonable mix of diet.			0.625				
24		I can drink enough water (2,000 ml) every day.			0.560				
28		I can maintain my dwelling environment naturally ventilated.			0.520				
16		I can wear loose-fitting, soft-textured clothing.					0.811		
15		I can maintain my personal hygiene.					0.758		
17		I can avoid using cosmetics that have caused allergies.					0.657		
13		During the active phase of the disease, I can try to avoid any type of physical trauma, such as scalds, burns, or abrasions.					0.609		
18	Except during the active phase of the disease, I can get appropriate exposure to sunshine.						0.453		
7	I can master the correct use method of topical medications.							0.717	
8	I can master matters needing attention of topical medications.							0.711	
31	I can take the initiative to learn about psoriasis health care and prevention in some way (e.g. formal internet, medical books, journals and magazines, etc.)							0.692	
6	I can go to a regular hospital to accept standardized treatment under the specialists, rather than blindly abusing prescriptions.							0.608	
1	I can take medications as prescribed and not reduce, increase, or discontinue them without authorization.								0.831
2	I can adhere to my usual medication schedule on special events such as holidays and outings.								0.827
4	I can reasonably schedule the type and timing of types of drugs as directed even if using the combination drugs.								0.695
Note: Factor loadings below 0.4 are not shown.									

**Table 4:** The standardized factor load, composite reliability and convergent validity

			UNSTD	*SE*	*Z*	*P*	STD	AVE	CR
Q33	<---	F1	1				0.704	0.560	0.910
Q34	<---	F1	1.056	0.086	12.218	^***^	0.802		
Q35	<---	F1	1.092	0.099	11.009	^***^	0.720		
Q36	<---	F1	1.177	0.096	12.233	^***^	0.803		
Q37	<---	F1	1.054	0.086	12.236	^***^	0.803		
Q38	<---	F1	1.205	0.096	12.58	^***^	0.827		
Q39	<---	F1	0.900	0.101	8.907	^***^	0.580		
Q40	<---	F1	1.079	0.099	10.937	^***^	0.715		
Q21	<---	F2	1				0.797	0.515	0.894
Q22	<---	F2	1.006	0.069	14.609	^***^	0.817		
Q23	<---	F2	0.857	0.075	11.435	^***^	0.673		
Q24	<---	F2	0.750	0.072	10.481	^***^	0.625		
Q25	<---	F2	0.940	0.083	11.313	^***^	0.667		
Q26	<---	F2	0.752	0.079	9.508	^***^	0.575		
Q27	<---	F2	0.970	0.073	13.315	^***^	0.761		
Q28	<---	F2	0.843	0.060	13.968	^***^	0.789		
Q13	<---	F3	1				0.587	0.636	0.894
Q15	<---	F3	1.290	0.116	11.087	^***^	0.952		
Q16	<---	F3	1.263	0.114	11.103	^***^	0.955		
Q17	<---	F3	1.089	0.112	9.703	^***^	0.760		
Q18	<---	F3	1.054	0.120	8.811	^***^	0.661		
Q6	<---	F4	1				0.746	0.645	0.877
Q7	<---	F4	1.199	0.080	14.948	^***^	0.906		
Q8	<---	F4	1.304	0.089	14.667	^***^	0.888		
Q9	<---	F4	0.912	0.088	10.312	^***^	0.643		
Q1	<---	F5	1				0.926	0.789	0.918
Q2	<---	F5	0.962	0.046	20.78	^***^	0.868		
Q4	<---	F5	0.924	0.044	20.839	^***^	0.870		

Abbreviations: AVE, average variation extraction value; CR, composite reliability; SE = Standard error; STD, standardized factor load; UNSTD, Unstandardized factor load; Z, regression weight estimate

^***^P < 0.001

**Table 5:** The correlation coefficients and discriminant validity

	AVE	Factor1	Factor2	Factor3	Factor4	Factor5
Factor1	0.560	**0.748**				
Factor2	0.515	0.709	**0.718**			
Factor3	0.636	0.582	0.709	**0.797**		
Factor4	0.645	0.621	0.669	0.715	**0.803**	
Factor5	0.789	0.645	0.618	0.592	0.732	**0.888**

Note: The value on the diagonal is the square root of AVE, the value of the lower triangle is the Pearson correlation coefficient between different dimensions

### Criterion validity

The General Self-Efficacy Scale was adopted as the criterion instrument to measure the criterion validity of the SMEQ-PSO. As a result of the correlation analysis, the correlation coefficient between the SMEQ-PSO and GSES was 0.407 (*P*<0.01).

### Reliability

As a result of reliability analysis, the Cronbach’s α coefficient for the total questionnaire was 0.930. The Cronbach’s α coefficient for self-efficacy of psychosocial adaptation, self-efficacy of daily life management, self-efficacy of skin management, self-efficacy of disease knowledge management and self-efficacy of disease treatment management were 0.898, 0.861, 0.817, 0.709 and 0.845, respectively. The total split-half reliability coefficient of the questionnaire was 0.952(*P* < 0.01).The total test–retest reliability coefficient of the questionnaire was 0.768 (*P* < 0.01).

## Discussion

An increasing body of research suggests that self-management efficacy is critical in improving the health outcomes of PSO patients, but there are few particular evaluation tools for assessing self-management efficacy among these patients [23, 35, 36]. Accordingly, it is essential to develop appropriate tool to identify and evaluate self-management efficacy among PSO patients. This study developed that the 28-item SMEQ-PSO is a reliable and valid self-report measure for assessing the self-management efficacy in PSO patients. This tool can be used to assess patients’ confidence in disease self-management and as an assessment tool for self-management interventions in clinical practice. By evaluating the self-management efficacy of patients with PSO, healthcare providers can identify the patients with low self-management and its weakness, as well as provide a basis for the follow-up implementation of effective intervention research and relevant health education.

### The practicability of the SMEQ-PSO

**Fig. 3:**
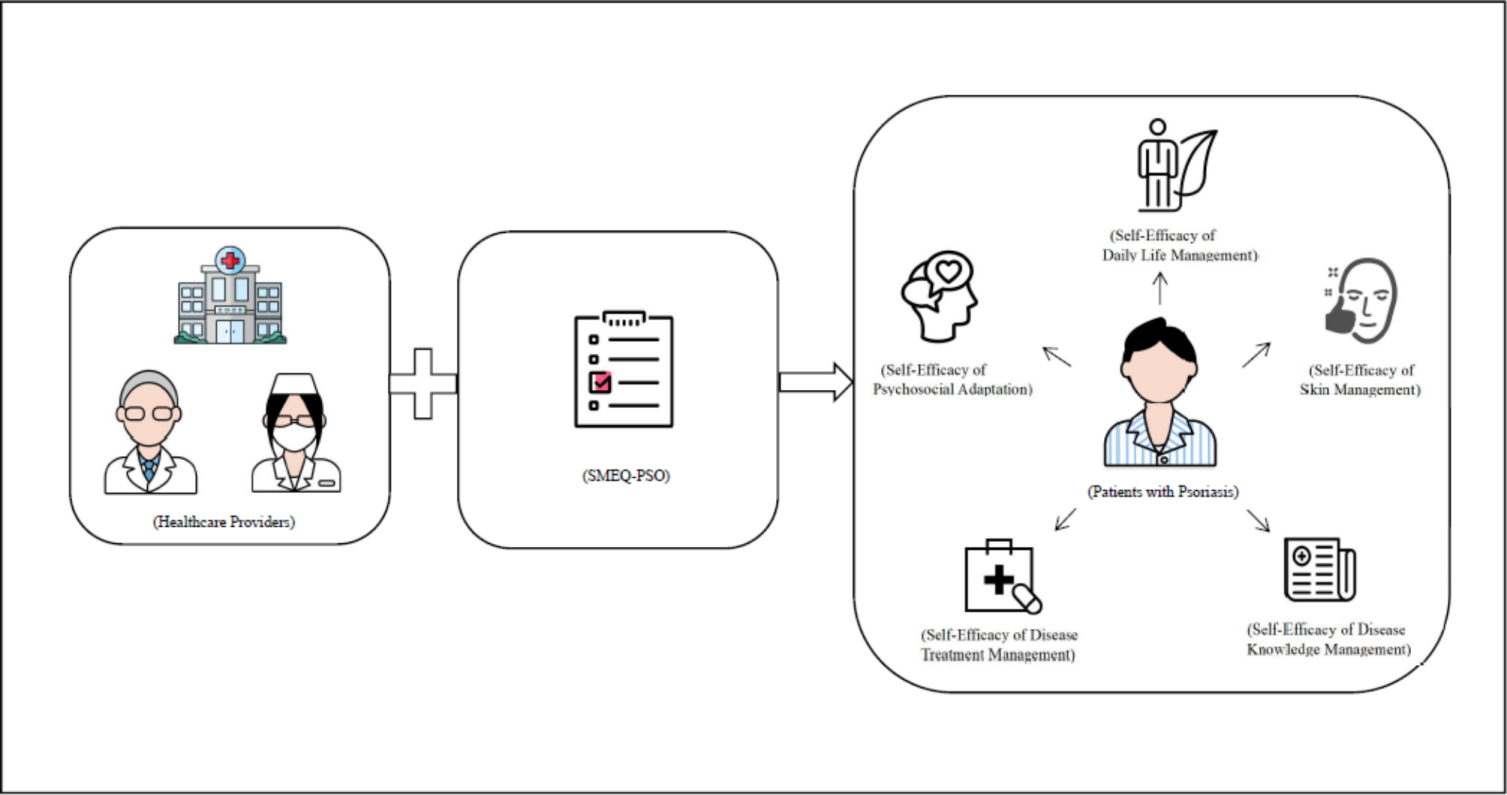
Application of the Self-Management Efficacy Questionnaire among Patients with Psoriasis (SMEQ-PSO) in clinical practice

The questionnaire developed in this study evaluated multiple aspects of PSO patients’ self-management efficacy, including self-efficacy of psychosocial adaptation, self-efficacy of daily life management, self-efficacy of skin management, self-efficacy of disease knowledge management and self-efficacy of disease treatment management. Our study took into account the main issues with current psoriasis management, which were PSO patients’ poor medication compliance, lack of attention to skin care, unhealthy lifestyle, and other concerns that needs to be resolved urgently [8, 37, 38]. These problems are reflected in the questionnaire. The content of the items was comprehensive and specific, containing not only reasonable and complete medical and lifestyle management assessment items, but also including psychosocial management content to achieve a comprehensive assessment of the overall self-management efficacy of patients with PSO. By combining self-management efficacy and the special features of psoriasis, the questionnaire overcomes the shortcomings of the universal scale due to its abstractness and low sensitivity. Additionally, the SMEQ-PSO can be understood easily, answered quickly and would be convenient for administering in a busy dermatology practice, which is to clearly and effectively evaluate the self-management efficacy of patients with PSO in disease treatment, daily life, and psychosocial aspects, as well as to understand the patients’ self-management demands. In conclusion, the SMEQ-PSO had a certain practicality in that it could thoroughly evaluate the level of self-management efficacy of patients with PSO. The application of the SMEQ-PSO in clinical practice is shown in Fig. 3.

### The scientificity of the SMEQ-PSO

In the process of compiling the questionnaire, this study is based on the self-efficacy theory, in conjunction with guidelines and expert consensus on PSO, and supplemented with items from other chronic disease self-management scales to ensure that the questionnaire has a scientific theoretical basis. Semi-structured interviews with PSO patients made the content of SMEQ-PSO closer to patients and clinical. Furthermore, the items were screened and refined through expert consultation and pre-survey. The specialists participated in the expert consultation have extensive theoretical research and practical expertise in the field, ensuring the questionnaire’s logic and integrity. The recovery rate for the two rounds of expert consultation questionnaires was 100%, indicating a high level of participation by experts. We used a variety of item analysis methods to analyze and evaluate and strictly screen the questionnaire items, and the research team had several discussions combined with the professional significance of the items to ensure rigor. As a result, the SMEQ-PSO developed in this study was highly scientific.

In terms of content validity, I-CVI and S-CVI were higher than the standard value, supporting that the questionnaire had appropriate content validity [30]. The five-factor structure derived from EFA adequately explains the overall variation. Additionally, CFA further supported the expected theoretical model, and the model fitting indexes were acceptable [39, 40]. Also, the questionnaire has good convergent validity and discrimination validity as evidenced by the proper AVE and CR values and the square root of AVE values being greater than the correlation coefficient between the related variables [31]. The aforementioned data clearly proved that the SMEQ-PSO has suitable construct validity. In criterion-related validity, the high correlation between the SMEQ-PSO and the GESE also demonstrated that the questionnaire has appropriate criterion-related validity. In reliability analysis, the Cronbach’s α coefficient for the total questionnaire was 0.930, and the Cronbach’s α coefficients for the factors ranged from 0.709 to 0.898. It showed that items within five dimensions generally were associated with each other, and the questionnaire has good internal consistency [34]. The total split-half reliability coefficient of the questionnaire was 0.952, with good homogeneity and intrinsic correlation between items [34]. The total test–retest reliability coefficient of the questionnaire was 0.768, indicating an appropriate stability of the questionnaire [34]. In a word, these proved that the questionnaire had good reliability. Our study has a number of limitations that need to be paid attention to and discussed. First, even though the study’s sample size satisfies the statistical standards, a sizable multicenter sample is still worthwhile taking into account to increase sample representativeness. Second, the research was conducted only in China. Cross-cultural validation studies are necessary. Lastly, the SMEQ-PSO was used to assess its predictive effectiveness as the emphasis of our future study.

## Conclusions

The 28-item five-factor SMEQ-PSO developed in our research showed good reliability and validity, which was confirmed in Chinese patients, and it could be used as an effective tool for clinical assessment and further research. It may also assist healthcare providers in assessing the level of self-management efficacy in patients with PSO and providing personalized interventions based on their individual circumstances to improve their quality of life.

## Data Availability

The datasets used and/or analysed during the current study are available from. the corresponding author on reasonable request.

## Abbreviations

PSO: Psoriasis
GBD: Global Burden of Disease
SME: Self-management efficacy
SMEQ-PSO: Self-Management Efficacy Questionnaire among Patients with Psoriasis
CR: Critical ratio
ITC: Item-total correlation
I-CVI: Content validity index of the item
S-CVI: Content validity index of the scale
EFA: Exploratory factor analysis
CFA: Confirmatory factor analysis
AVE: Average variance extracted
CR: Composite reliability
GSES: General Self-Efficacy Scale
KMO: Kaiser–Meyer–Olkin
χ2/df: Chi-square degree of freedom
RMSEA: Root mean square error of approximation
GFI: Goodness-of-fit index
CFI: Comparative fit index
IFI: Incremental fit index
PNFI: Parsimonious normed-of-fit Index
PGFI: Parsimonious goodness-of-fit index
